# Sensory Agreement Guides Kinetic Energy Optimization of Arm Movements during Object Manipulation

**DOI:** 10.1371/journal.pcbi.1004861

**Published:** 2016-04-01

**Authors:** Ali Farshchiansadegh, Alejandro Melendez-Calderon, Rajiv Ranganathan, Todd D. Murphey, Ferdinando A. Mussa-Ivaldi

**Affiliations:** 1 Sensory Motor Performance Program, Rehabilitation Institute of Chicago, Chicago, Illinois, United States of America; 2 Department of Biomedical Engineering, Northwestern University, Evanston, Illinois, United States of America; 3 Department of Physical Medicine & Rehabilitation, Northwestern University, Chicago, Illinois, United States of America; 4 Department of Kinesiology, Michigan State University, East Lansing, Michigan, United States of America; 5 Department of Mechanical Engineering, Northwestern University, Evanston, Illinois, United States of America; 6 Department of Physiology, Northwestern University, Chicago, Illinois, United States of America; Imperial College London, UNITED KINGDOM

## Abstract

The laws of physics establish the energetic efficiency of our movements. In some cases, like locomotion, the mechanics of the body dominate in determining the energetically optimal course of action. In other tasks, such as manipulation, energetic costs depend critically upon the variable properties of objects in the environment. Can the brain identify and follow energy-optimal motions when these motions require moving along unfamiliar trajectories? What feedback information is required for such optimal behavior to occur? To answer these questions, we asked participants to move their dominant hand between different positions while holding a virtual mechanical system with complex dynamics (a planar double pendulum). In this task, trajectories of minimum kinetic energy were along curvilinear paths. Our findings demonstrate that participants were capable of finding the energy-optimal paths, but only when provided with veridical visual and haptic information pertaining to the object, lacking which the trajectories were executed along rectilinear paths.

## Author Summary

Recent studies have shown that when learning novel dynamics in the context of reaching movements, people often ignore energetic optimality in favor of Euclidean geometric optimality, preferring rectilinear paths over mechanically optimal trajectories. Although an explanation could be that sensory-motor coordination ignores energetic cost, another possibility is that different sensing modalities need to be in agreement before the brain will optimize energetic cost during motion. We provide evidence for this latter perspective, by showing that when provided congruency and consistency of visual and haptic feedback, participants take into account both geometric and mechanical properties of a manipulation task. However, when visual and haptic feedback are inconsistent, participants revert to the rectilinear paths seen in previous studies. We conclude from these observations, that when transporting an external object, sensory agreement between vision and touch guides the optimization of the kinetic energy exchanged during movement between the arm and the object.

## Introduction

One of the most established findings in planar multi-joint reaching movements is that hand trajectories tend to be executed along straight paths with a bell-shaped velocity profile [1–3]. Given that there are theoretically infinite paths that the hand could take for reaching from one point to another, the presence of this consistent feature in reaching movements has been used to suggest that the nervous system chooses this solution because it is “optimal” in some way. Mathematical optimization has been considered as an appealing principle to explain observed biological movements. Optimization requires an objective function, or cost, that includes the quantities being minimized. The choice of cost has received much attention in the study of neural information processing, in particular, by the motor system [4–8]. The components of an objective function generally fall into two main types: kinematic and dynamic. While the former relates only to the geometry of motion, the latter relates to the forces that cause the motion. Despite fundamental differences between the two types, objective functions consisting purely of one or the other have been similarly successful in predicting data obtained from unperturbed planar reaching movements. Adaptation studies have attempted to distinguish between kinematic and dynamic costs by introducing perturbations to these movements. It has been shown [9, 10] that in the presence of kinematic perturbations, participants chose to move the hand along curved paths so as to produce visually straight trajectories. Similarly, under the dynamic perturbation caused by forces depending upon the velocity of the hand, subjects learned to recover straight hand trajectories through repeated practice of reaching movements [11]. More recently, to evaluate if mechanical energy costs play a role in motor learning, a custom force field was designed in a way that the path of minimum mechanical energy was substantially different from the straight path [12]. Under this situation, participants returned to straight line reaches even after they experienced moving along the energy optimal path. These studies suggest that the tendency to move the hand on a straight line in planar movements is strong and persistent, arising under a variety of dynamic and kinematic perturbations, and indicate that the kinematic costs are either necessary [13] or sufficient [14] components of the cost function.

However, these previous studies were typically focused on unconstrained movements of the hand in free space and when a force field disturbed these movements. In the majority of earlier studies a cursor was used as a visual image of the system under control [11, 12, 15]. This representation makes all spatial directions visually equivalent and moving the cursor on a straight line appears to be an economical approach. Moreover, the haptic feedback (i.e. the contact forces experienced during movements) were predominantly in the form of force fields. In these experiments, there were no features in the visual scene or in the shape of the cursor that could be associated with the forces experienced by the subjects. We hypothesized that this dissociation between sensory modalities elicits a compensatory strategy where movements are channeled to restore the kinematics of the unperturbed hand motion. Conversely, congruence between feedback modalities, representing the action upon an identifiable external object is expected to result in energy efficient strategies. In this case, motor learning leads to a progressive optimization of the energetic costs of movements rather than a process towards recovering a straight invariant trajectory. Depending on the object's dynamics, the resulting trajectory may systematically and substantially deviate from the straight line. Therefore to test this prediction, we used an object manipulation task where there is a well-defined relation between the visual and haptic feedback.

## Results

Participants executed goal-directed reaching movements in the horizontal plane while holding the end point of a virtual planar double pendulum in the absence of gravitational effects. The choice of the double pendulum was motivated by the fact that the energy-optimal trajectories for moving this object were along curved paths, allowing us to tease apart the relative importance of kinematic and dynamic costs.

**Fig 1:**
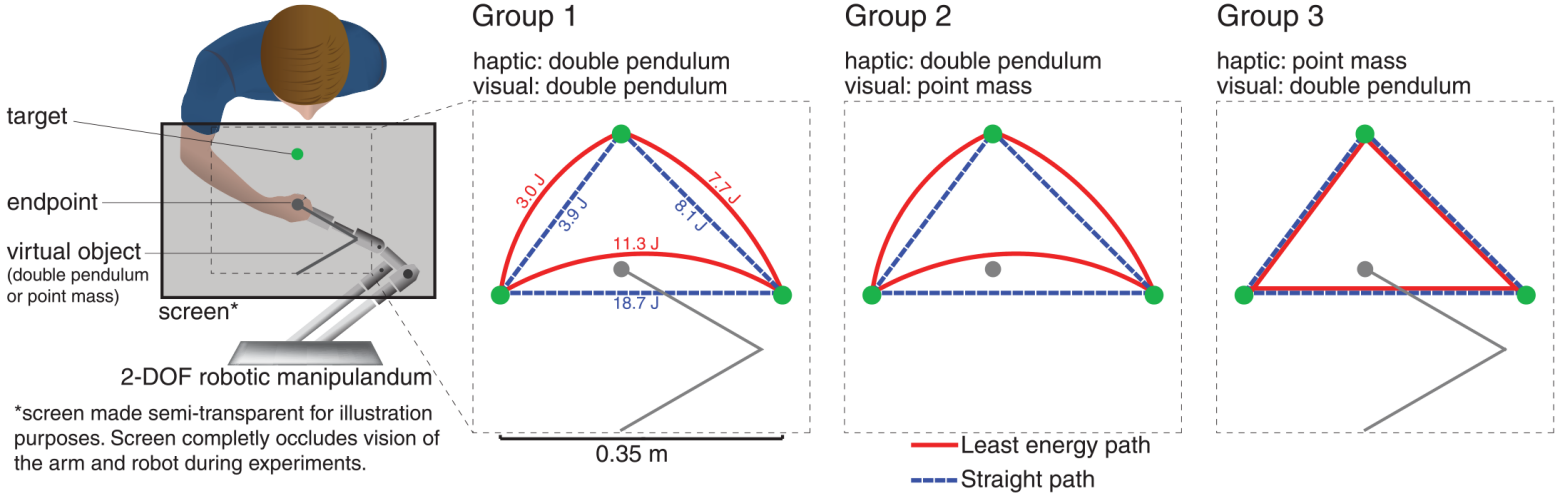
Experiment design. Straight and least energy paths between each pair of targets in the task space coordinate frame.

The energy-optimal paths for moving this system were calculated as follows. The total energy for this system consists only of the kinetic term. The path of least kinetic energy between any two double pendulum configurations is a solution to a two-point boundary value problem—leaving the initial and final velocities as free variables- of the unforced system and it is generally curved in shape (Fig 1). However, this path is a purely geometric quantity and from a control perspective, it is not an admissible solution because the velocity requirements are not satisfied. To find an admissible solution we used optimal control theory with the only running cost of effort, defined as the force being applied to the object. Expectedly, the path of minimum energy and the effort optimal trajectory had similar shapes (see S1 File). The energy (mechanical work) that is acquired by the object upon point-to-point maneuvers was calculated by

$$E=\int_{0}^{T}|Fv|dt$$ (1)

where *F* represents the force applied to the object, *v* is the velocity of the hand and *T* is the movement time. The energy that was required to move along the straight path and the path of least energy between each pairs of targets is included in Fig 1. These values are obtained from a minimum jerk trajectory with the movement duration of 1 sec.

Participants were randomly divided in three groups. All received both visual and haptic feedback. Participants in Group 1 (n = 8) received veridical visual and haptic information of the double pendulum. For Group 2 (n = 8), only the visual feedback was manipulated. Participants in this group were presented with a circular cursor representing the moving extremity of the pendulum. They did not see the linkage but the haptic feedback corresponded to the entire mechanism. Participants in Group 3 (n = 8) could see the entire linkage while the haptic information was manipulated so as to emulate the isotropic inertia of a point mass. In this scenario, our hypothesis made an explicit prediction on the trajectory formation: If participants were provided with full vision of the manipulated object together with a congruent haptic feedback (Group 1), they would integrate the geometric structure with interaction forces to converge to the curvilinear paths of minimum energy. In contrast, if participants only received visual feedback of the endpoint (Group 2), or haptic feedback corresponding to point-mass dynamics (Group 3), then movements would be executed along rectilinear paths because of the lack of consonance between the sensing modalities.

**Fig 2:**
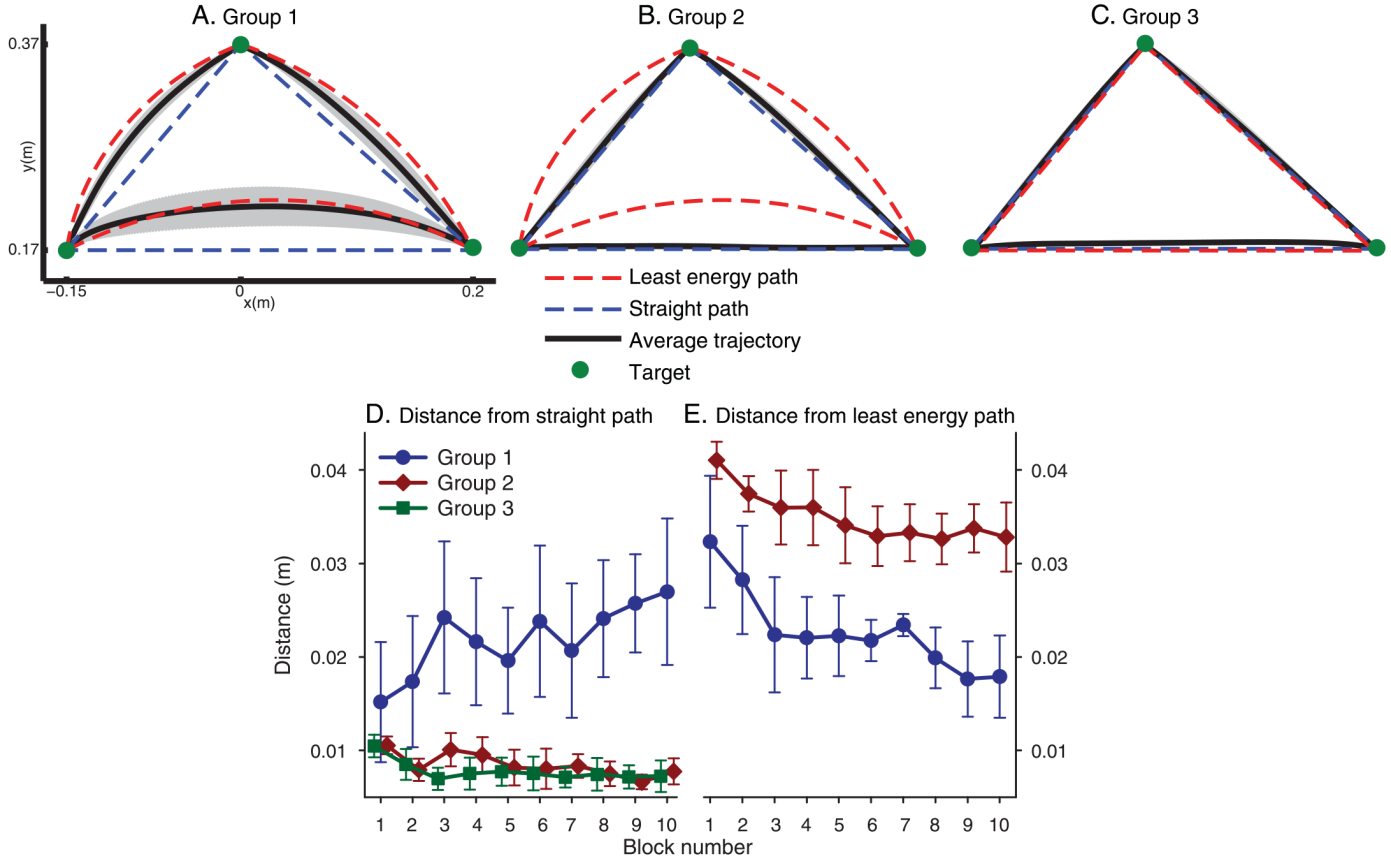
Results. (A) Average trajectories of participants in Group 1 (double pendulum vision / double pendulum haptics) during the last block. (B) Average trajectories of participants in Group 2 (point mass vision / double pendulum haptics) during the last block. (C) Average trajectories of participants in Group 3 (double pendulum vision / point mass haptics) during the last block. (D) Average discrete Fréchet distance from the straight path. (E) Average discrete Fréchet distance from the path of least kinetic energy. For Group 3, the straight path and the path of least energy were the same. Error bars and shaded area represent 95% confidence level.

For each trial, movement initiation and termination were identified using 10% of peak velocity threshold. Participants in all the three groups started the experiment by moving along straight line trajectories. However, the trajectory divergence between Group 1 and the remaining groups started after the very first few trials. We found that with practice, all participants that received congruent visual and haptic feedback progressively moved towards producing curved trajectories that were similar to the path of minimum energy. This gradual adjustment suggests that the problem of finding the energetically optimal trajectory was solved via gradient descent beginning from the straight line trajectory typical of the freely moving hand, In contrast, all participants that were presented with incongruent feedback continued to move along rectilinear paths (Fig 2). We quantified the similarity of executed trajectories to both straight line and least energy paths using discrete Fréchet distance (DFD) [16]. The Fréchet distance between two curves is the minimum cord-length that is sufficient to join two points traversing each curve with arbitrary speeds without backtracking. Intuitively, imagine a dog walking along one curve and the dog’s owner walking along the other curve and they are connected by a leash. Both can change the speed and even stop at arbitrary positions with arbitrary durations but neither are allowed to move backwards. The Fréchet distance between the two curves is the length of the shortest leash that connects the man to the dog at all time. One-way ANOVAs on DFD from the straight path and DFD from the least energy path during the last block revealed a significant group effect on both distances. Dunnett’s post-hoc tests showed that Group 1 was significantly further from the straight path than the two other groups (p <0.01). Similarly Group 1 was significantly closer to the path of minimum energy compared to Group 2 (p <0.01).

One feature in the result is that although the participants in Group 1 show greater curvature, they did not completely converge to the energy efficient path. We speculate that this may be due to the fact that participants in Group 1 did not have any explicit knowledge about the mechanical properties of the object and the geometric shapes of the effort optimal trajectories. They derived these trajectories solely based on the sensory information. Therefore, considering noise and model uncertainties in sensory transduction and neural computation, they were expected to move at larger distances from the paths of minimum energy and exhibit greater variability in their movements in comparison with participants in the remaining groups who moved along straight paths and had explicit kinematic plan for executing their movements.

## Discussion

Our results suggest that when learning novel dynamics, if the visual representation is a cursor or a shape that is indicative of isotropic dynamical structure, this impoverished representation provides a strong bias towards Euclidean representations of the configuration space, where all directions are equivalent and straight lines are the natural geometrical paths for joining two points. In this situation, participants experience a mismatch between expected and sensed forces under the assumption that they are moving the arm in free space. This mismatch between sensory information triggers a compensatory strategy where subjects fight the force field to recover the straight unperturbed trajectory. However, if subjects are provided with any visual information suggestive of an external object being manipulated, with non-Euclidean dynamical structure to begin with (because of the non-isotropic, position dependent inertia tensor at the contact point), then they attribute the haptic feedback to the visual image and with practice try to develop a representation of the object’s configuration space. This harmony between sensing modalities promotes a control policy that requires less effort to perform the task. Work on remapping finger movements has highlighted that when subjects learn a novel task of manipulating a kinematic chain by continues finger motions, movement trajectories are formed along the geodesics (i.e., paths of minimum length) corresponding to the geometrical structure of that object [17]. Subjects in Group 1 and 3 were both provided with the same visual feedback but at the end of the experiment they moved along different paths, each corresponding to the path of minimum energy of the object that they were manipulating (double pendulum haptics vs point mass haptics). This result confirms that curved trajectories observed in Group 1 is not a solution to the kinematic problem but it is a progressive optimization of the energy exchanged with the object. Here, effort was defined as the force that subjects applied to the system to move it between target positions. Although we did not measure the metabolic effort (i.e. the physiological energy cost), a recent study found that indeed the metabolic effort is reduced during force field adaptation [18].

The conclusions of previous studies on energy optimization in human motor control are mixed. Some studies suggest that the motor system is capable of minimizing the energetic costs of free limb movements both in arm reaching movements [19] as well as locomotion [20], while other studies of learning novel dynamics suggest that the motor system does not take into account the energy when executing movements [12, 14]. When moving a limb or manipulating an object the energy optimal solution depends on the mechanical properties rather than the visual representation. However, there is considerable amount of evidence that the movement control policy and consequently trajectory formation both in free reaching movements and in object manipulation depends remarkably on the visual feedback.

Straight line trajectories are found typically in studies of free arm movements when the sight of the arm is obstructed and the subjects are presented with a cursor. However, it has been shown that trajectories of the free reaching movements of congenitally blind and even blind folded individuals to haptic targets are more curved than movements made by subjects to the same target positions under visual guidance [21, 22]. Similarly, subjects performed curved motions when they were instructed to reach to physical targets with their arm rather than reaching with a cursor to a virtual target [19]. These studies suggest that in free limb movements, humans can flexibly alter movement behavior between geometric and energetic optimally depending on the feedback. In contrast to free movements, moving in a force field provides an additional challenge to the nervous system because in this case, the effort optimal trajectory not only depends on the mechanics of the body, but also on the dynamical properties of the field. It has been demonstrated that the representation of the dynamics of a manipulated object also depends on the visual representation [23] and that only specific and meaningful visual cues can promote proficient switching between different mechanical tasks [24]. Recent studies on learning novel dynamics reported that the motor system ignores energetic costs in favor of geometric optimality. However subjects in these studies were exposed to a force field with the representation of a cursor [12] or with no visual feedback [13]. In the latter study the visual feedback (cursor) was provided only at the beginning and at the end of each trial. Here, we showed that in object manipulation, the visual motion of the object resulting from an applied force is a critical piece of information for the brain to represent the dynamics. Given our finding that subjects learned to minimize the energy transfer with an object having anisotropic position-dependent inertial properties, we observe that the most common objects being transported by our hands have isotropic position-independent translational inertias. They are therefore characterized by straight-line kinetic energy geodesics when moving on the horizontal plane. Thus, we speculate that the tendency to perform straight-line planar movements of the hand may be a baseline behavior emerging from the experience of transporting such objects while optimizing the energy exchanged with them. It has been shown [19] that the arm trajectory in a 3D pointing movement is along the geodesic path that is obtained through the minimization of the kinetic energy on the configuration space of the arm. The Euler- Lagrange equations are equivalent to the equations of geodesic motion on a Riemannian manifold. We extended the computational model in [19] to learning novel dynamics, we showed that trajectory formation hinges on the consistency between feedbacks representing the system under control, and how feedback variations can lead to remarkably different behaviors. The demonstrated results provide insights into studies on adaptation, effort minimization and object manipulation by the human motor system.

## Materials and Methods

Twenty four right-handed volunteers (12 female) participated in the experiment. All participants were neurologically intact and had no prior knowledge of the experimental procedure. The study protocol was approved by Northwestern University’s Institutional Review Board and all the participants signed an informed consent form. Participants were positioned in front of a horizontal mirror and held the handle of a planar, two degree of freedom robotic manipulandum with their right hand. The mirror prevented the participant’s view of their hand and the robot. A LED monitor was positioned above the mirror with the same vertical distance as the distance between the robot and the mirror. This setup caused the visual information to appear at the same height as the hand. The display was calibrated so that the visual feedback of the hand was overlaid on its true position.

Participants performed goal-directed reaching movements to three targets (diameter = 3 cm). Targets were presented in a block structure, with randomized order within each block. After reaching to each target, participants maintained the position for 500 ms before the next target appeared. The experiment consisted of 10 blocks and in each block participants performed 48 reaching movement (16 reaches per target). Participants could rest between blocks. During all these reaching movements, the manipulandum was either connected to the endpoint of a virtual double pendulum with the mechanical properties that are listed in Table 1 or a virtual 15 kg point mass, by means of a virtual spring-damper (K = 2200 N/m, B = 65 N.s/m). Position and velocity of the manipulandum handle were computed from instrumented encoders at the frequency of 1 kHz to provide haptic feedback of the forces resulting from moving the double pendulum or the point mass. The manipulandum was equipped with electric motors with the peak torque of 82 Nm. However, the maximum torque that the robot was asked to generate in the fastest recorded trial in this experiment was about 15 Nm. Data were recorded at the rate of 100 Hz. Participants were randomly divided into three groups of equal size (n = 8 per group): Group 1, where participants received both the visual and haptic feedback of the double pendulum. Group 2, where participants received the visual feedback of the moving extremity of the pendulum that was held in their hand, in form of a circle (diameter = 1.5 cm) and the haptic feedback of the double pendulum. Group 3, where participants received the visual feedback of the double pendulum with haptic feedback of the point mass. One of the participants in Group 3 revealed that he was familiar with the purpose of the study and was replaced by another participant.

**Table 1:** Mechanical properties of the virtual double pendulum.

Mass	*m*_1_ = *m*_2_ = 10*kg*	Inertia	*I*_1_ = *I*_2_ = .4*kg*.*m*^2^
Length	*l*_1_ = *l*_2_ = .2*m*	Center of mass	*c*_1_ = *c*_2_ = .1*m*

## Supporting Information

S1 File Comparison between path of minimum kinetic energy and effort-optimal trajectory. (PDF)

S1 Data All experimental data used in this work. (ZIP)
