# Supplementary material for: Sensory Agreement Guides Kinetic Energy Optimization of Arm Movements during Object Manipulation
Source: PLoS Comput Biol. 2016 Apr 1;12(4):e1004861. doi: 10.1371/journal.pcbi.1004861 (PMC4818082; doi:10.1371/journal.pcbi.1004861)
Supplement: S1 File — (PDF) [file pcbi.1004861.s001.pdf]

S1: Comparison between path of minimum kinetic energy and effort-optimal trajectory

The second order nonlinear equations of motion for a double pendulum can be derived using the Euler-Lagrange equations and expressed in matrix form as

$$M(q)\ddot{q}+C(q,\dot{q})\dot{q}=u \tag{s1}$$

Where $M(q)$ is the inertia matrix

$$\begin{aligned}
M_{1,1}(q) &= I_1+I_2+m_2l_1l_2\cos(q_2)+\frac{1}{4}\left(m_1l_1^2+m_2l_2^2\right)+m_2l_1^2\\
M_{1,2}(q) &= M_{(2,1)}(q)=I_2+\frac{1}{2}m_2l_1l_2\cos(q_2)+\frac{1}{4}m_2l_2^2\\
M_{2,2}(q) &= I_2+\frac{1}{4}m_2l_2^2
\end{aligned} \tag{s2}$$

$C(q,\dot{q})$ is the Centripetal/Coriolis matrix

$$C(q,\dot{q})=\begin{bmatrix} -\frac{1}{2}m_2l_1l_2\sin(q_2)\dot{q}_2 & -\frac{1}{2}m_2l_1l_2\sin(q_2)(\dot{q}_1+\dot{q}_2) \\ \frac{1}{2}m_2l_1l_2\sin(q_2)\dot{q}_2 & 0 \end{bmatrix} \tag{s3}$$

u represents generalized forces on the system. m_i, I_i, l_i and q_i are the mass, rotational inertia, length and joint angle of the i-th link, respectively. The center of mass for each link is considered to be in the middle.

For the unforced system ($u=0$), the path of minimum kinetic energy can be derived by solving a two point boundary value problem where the boundary conditions are set to be the system's initial and final joint angles and leaving the joint velocities as free variables. This path is a purely geometrical quantity and from a control perspective is not admissible because it does not require the initial and terminal velocities to be zero. To find an admissible solution we used optimal control theory with the only running cost of effort, defined as the force being applied to the object. From equation (s1), we can compute the forward dynamics and write the equations in state space form

$$\dot{x}(t)=f\left(x(t),u(t)\right),\ \ x(0)=x_0 \tag{s4}$$

Where $x=(q_1\ q_2\dot{q}_1\dot{q}_2)^T$. The cost function is defined as

$$J\left(x(t),u(t)\right)=\frac{1}{2}\left[\int_0^{t_f}u(t)^T Ru(t)dt+\left(x(t_f)-x_d(t_f)\right)^T P\left(x(t_f)-x_d(t_f)\right)\right] \tag{s5}$$

The integrand part only includes the control cost without having a desired reference trajectory. $x_d(t_f)$ is the desired state vector at the end of the movement. R and P are positive definite

weighting matrices with $R = \begin{bmatrix} 0.001 & 0 \\ 0 & 0.001 \end{bmatrix}$ and $P = I_{4\times4}$. The final time $t_f = 2\,sec$, corresponds to the average movement time for subjects in this study. As shown in figure S1, the obtained effort-optimal trajectories are very similar to the paths of minimum kinetic energy. It is useful to note that the shape of trajectories are robust to changes in the weighting matrices.

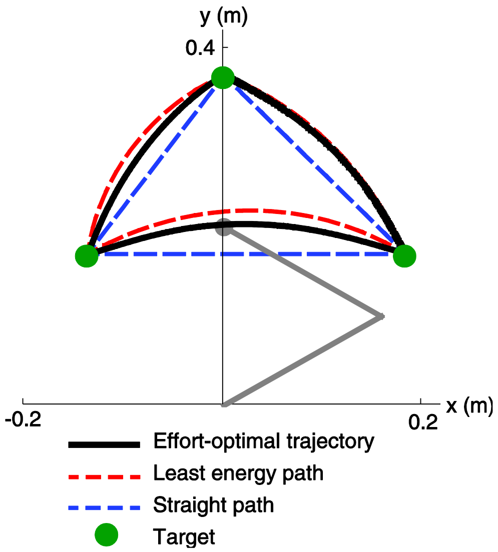

Figure S1. Comparison between path of minimum energy and optimal trajectory with a quadratic running cost of effort and a terminal cost.
